# Comparative Analysis of *Petaurus* Cryptic Species of ‘Sugar Glider’ from Australia and New Guinea Using 3D Geometric Morphometrics

**DOI:** 10.3390/ani14243680

**Published:** 2024-12-20

**Authors:** Meagan Powley, Katarina Mikac

**Affiliations:** Environmental Futures, School of Science, University of Wollongong, Wollongong, NSW 2522, Australia

**Keywords:** geometric morphometrics, sugar glider, population divergence, evolutionary adaptation, isolation effects, geographic variation

## Abstract

The description of the *Petaurus* sp. has been ongoing since first described and especially within the last fifteen years. Recently from the known *P. breviceps* species, three subspecies have been recognized at the species level. Two of these species, *P. notatus* and *P. ariel*, have been geographically described throughout Australia; however, the northeast Queensland labeled *P. breviceps* remain unrevised. A third species, *P. papuanus*, is located throughout New Guinea and is associated with at least one divergence event to Australia. Our research used geometric morphometrics and linear measurements to compare known Australian species with specimens from Cape York and New Guinea. No significant shape difference was found between the known specimens of *P. notatus* and Cape York specimens. Our findings support an earlier identification of *P. notatus (P. longicaudatus)* at this location. Significant shape variation was found between Cape York specimens and New Guinea, indicating no association with the New Guinea species. Significant shape changes were also found in island specimens when compared with the mainland New Guinea specimens, which was likely a result of their isolation and accelerated evolutionary responses to novel environments. No significant shape change was found for mainland New Guinea specimens, which was likely associated with overlapping distribution and hybridization.

## Simple Summary

Recent work on *Petaurus* species has recognized three subspecies of *P. breviceps* as distinct species. Two of these, *P. notatus* and *P. ariel*, are found in Australia, although the population in northeastern Queensland labeled as *P. breviceps* remains unreviewed. The third, *P. papuanus*, is found in New Guinea and likely dispersed to Australia through at least one divergence event. Our study used geometric morphometrics and linear measurements to compare known Australian species with specimens from Cape York and New Guinea. We found no significant shape differences between *P. notatus* and Cape York specimens, aligning with an earlier identification of *P. notatus* (previously *P. longicaudatus*) in this area. Significant shape variation was observed between Cape York and New Guinea specimens, suggesting no association to the New Guinea species. Furthermore, notable shape differences were detected between island and mainland New Guinea specimens, which was possibly due to their isolation and rapid evolutionary adaptation to distinct environments. In contrast, mainland New Guinea specimens showed no significant shape differences, which may result from overlapping distributions and hybridization among populations.

## 1. Introduction

The scientific naming of animals has been ongoing since the mid 1700s and in Australia and the surrounding islands since 1785. This process builds upon previous research by authors to provide clarity and further resolution of the taxonomy each species. The first Australian species to be scientifically described was the Eastern Ring-tailed Possum (1785), the Eastern Grey Kangaroo (1790) and the Yellow-bellied Glider (1791). What followed was a rapid increase in the number of Australian species’ scientific description, and by 1900, 230 species of native animals were described [1]. Throughout more than two centuries of taxonomy of Australasian fauna, some animals were more completely described, such as monotremes, bandicoots, seals and introduced species; however, other mammalian species remain unresolved. Diprotodontians, chiropterans, rodents and dasyurids have been partially resolved, especially since the use of genetic material initiated late in the 20th century, but the scientific description of these groups is still deficient [1]. Animals in remote or difficult to access habitat are a greater challenge to sample and formally identify. Increased numbers of species identified as endangered or threatened due to introduced predators and anthropogenetic modifications to habitat have heightened the urgency to describe Australian fauna [2]. The conservation and management of species based on known geographic distribution with the most informed taxonomic name is essential [3].

The *Petaurus* genus includes nine species of gliding marsupials, and for *P. breviceps*, there are currently four subspecies identified from Australia, New Guinea and the surrounding islands; *P. b. breviceps* (limited Australian east coast), *P. b. biacensis* [4] (Biak Island), *P. b. papuanus flavidus* [5] (western division New Guinea) and *P. b. papuanus tafa* [5] (eastern ridge of central New Guinea) [1,2,6]. The New Guinea Glider, *P. papuanus* [7] is a recent species recognized throughout New Guinea [2]. This cryptic complex of nocturnal gliding marsupials found in the canopy has likely constrained resolution of the taxonomy for *Petaurus*. Attempts to determine the taxonomy of the *P. breviceps* has been ongoing since first it was described [8], and efforts have intensified especially in the last thirty years [2,9,10,11,12,13]. A recent taxonomic review in Australia recognized two subspecies of *P. breviceps* as a separate species: namely, *P. ariel* [14] and *P. notatus* [9,15]. The authors reviewed phylogenetic and morphological data to resolve some uncertainty around the inland and northern Australian species. The geographic distribution for these species is described for *P. ariel* in the central–north of the continent, while it is also west of the Great Dividing Range and throughout the southeast and Tasmania (introduced) is *P. notatus*. The remaining narrow coastal range for P. breviceps has been considerably reduced to east of the Great Dividing Range. Resolution has not been determined for the northeastern *Petaurus* in Australia. However, an early description by Smith (1973) described the distribution of *P. b. longicaudatus* (now recognized as *P. notatus*) as inhabiting the northeast of Australia. The morphological characteristics of *P. breviceps* provided the formative description for populations of *P. breviceps* throughout Australia and New Guinea and early taxonomic description [6].

Early allozyme analysis provided evidence for the genetic difference between species throughout New Guinea and Australia previously described only with morphological variation. A marked population structure was found in species in the regions of Tifalmin, central New Guinea and Karkar Island, north of New Guinea [12]. However, other allozyme results did not correspond to the morphologically described groups. The molecular phylogenetic analysis (ND2 and 12S) for Australo-Papuan possums did not find clear evidence for monophyletic resolution and identified conflicts with morphological data [11]. Evidence for monophyletic resolution for Petauridae was also not well supported. Phylogenetic relationships were examined within the genus and found that currently recognized species formed distinct monophyletic clades. From *P. breviceps*, seven distinct clades were identified (ND2, ND4 and ω-globin gene) with conflicting geographic distribution compared to existing morphologically defined species [10]. There was evidence to support at least one dispersal event from New Guinea to Australia likely after the divergence between *P. breviceps* and *P. norfolcensis* but before the divergence of species from *P. breviceps* (~3.5–8.4 mya) [10]. Evidence for clarification for clades in Tifalmin and Karkar Island was not resolved.

Characteristics of live specimens and skull measurements were previously used to morphologically distinguish species by location; however, a geometric morphometric comparison of cranial shape has not been attempted for *P. breviceps* in northeastern Australia and New Guinea. Using homologous 3D landmarks of the cranium and by removing rotation, location and scale in a Procrustes superimposition, a dataset that compares only information about the shape of the specimens is developed [16,17,18]. Geometric morphometrics has proven effectual at differentiating shape variation for taxonomic purposes when compared to the use of only examining linear measurements [19]. Many species including rodents [20,21], bats [22,23,24], and small Australian marsupials [25] have used geometric morphometrics to contribute to taxonomical resolution in similar sized mammals. Evidence indicates that using landmarks that concentrate on the functional regions of the cranium (zygomatic bones, rostrum and braincase) when compared with those regions under a reduced functional constraint (middle ear to foramina of the ventral braincase) are more likely to reveal strong functional selection [26]. Strong functional selection as a result of population isolation, endemic habitat and dietary conditions are associated with taxonomic distinction [27].

The aim of this research was to review specimens of *P. breviceps. P. notatus* and *P. papuanus* available in museums throughout Australia where the provenance of the specimen is from a region identified by Malekian et al. (2010) [10] as a clade (NG1–NG5) or other region in New Guinea [6]. These specimens were compared with specimens in the north-east geographical region in Australia to examine whether morphological variation is found between the New Guinea clades and the Australian northeast specimens.

## 2. Materials and Methods

**Figure 1:**
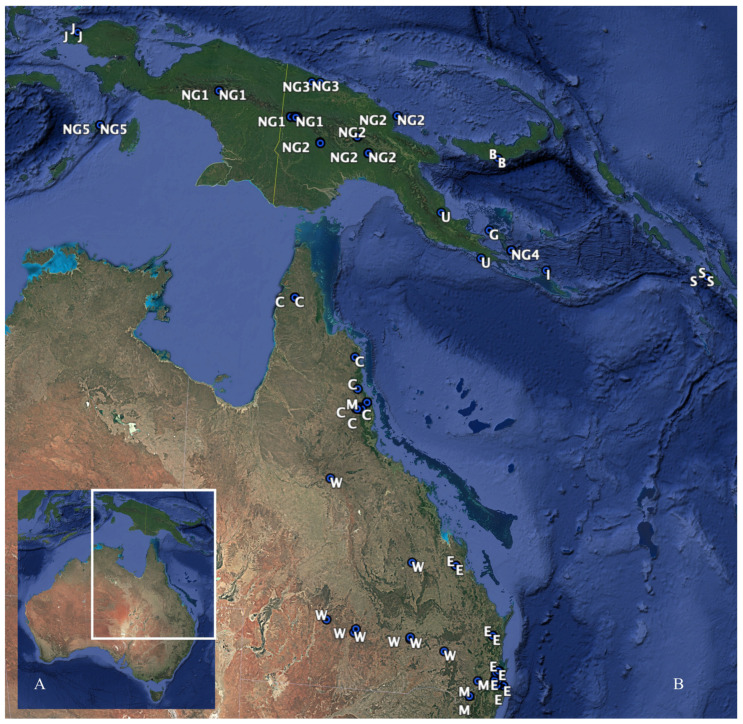
Location of specimens used from Queensland and New Guinea. (**A**) Location of Australia and New Guinea and white inserted box indicating study region. (**B**) Enlarged insert from (**A**) for specimen location. Multiple specimens were reviewed from some locations particularly in New Guinea indicated by green dot and white letters indicating region name described in Cremona et al. (2020) [9] and Malekian et al. (2010) [10]. (Image developed using Google Earth Pro (2022) 7.3.6.9345 (64-bit) Data SIO, NOAA, US. Navy, NGA, GEBCO Image Landsat/Copernicus).

Specimens analyzed in this study were sourced from Australian museum collections and allocated origins based on earlier research, other locations in the New Guinea region and Queensland locations. Using complete adult *P. breviceps*, *P. notatus* and *P. papuanus* skulls, 82 3D craniums models and 96 skulls were measured. Skulls were analyzed from the following institutions: Australian Museum 59, South Australia Museum 1, Museums Victoria 1 and Queensland Museum 35. Adult skulls were determined by the identification of the complete eruption of the permanent molars [9]. Specimen location and sex were obtained from museum records. New Guinea specimens were allocated to NG1 (Bundi and Gali, central–west New Guinea Male *n* = 4 Female *n* = 2), NG2 (Waro, Namosado, Tifalmin, Noru and Yuro, central–east New Guinea, Male *n* = 11 Female *n* = 17 unknown *n* = 1), NG3 (Wigote and Mt Sulen, north–central coast New Guinea, Male *n* = 1), NG4 (Normanby Island, Male *n* = 2 Female *n* = 4), NG5 (Kai Island, Male *n* = 3), and B (New Britain Island, Unknow *n* = 2), G (Goodenough Island, Male *n* = 1), I (Misima Island, Male *n* = 1), J (Irian Jaya, Male *n* = 1 Female *n* = 2), S (Solomon Islands, unknown *n* = 3), and U (southeast corner Papua New Guinea, Female *n* = 1 unknow *n* = 1). Australian specimens were allocated to C (Cape York region, Male *n* = 4 Female *n* = 4), E (east coast northern Queensland, Male *n* = 3 Female *n* = 7), M (region of Great Dividing Range (GDR) Male *n* = 4 Female *n* = 5) and W (West of GDR, Male *n* = 3 Female *n* = 6) (Figure 1).

A SOL PRO 3D Scanner (Scan Dimension, Global Scanning Denmark) was used to collect cranium scans. Craniums were scanned in a standard anatomical position using a 360° pass in the “High Accuracy” setting in the near lens position. Scans were processed and aligned in SOL PRO Viewer 2.0.0, and the final meshed object was used for landmarking. Landmarking the 3D model was guided by textured layers in Slicer V5.4.0 [28].

**Figure 2:**
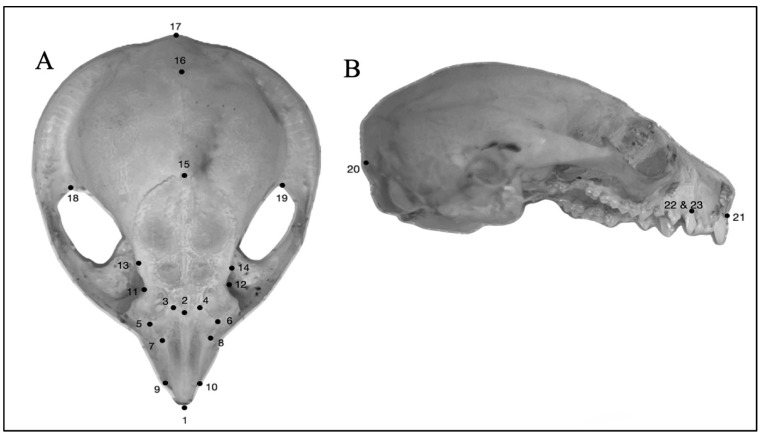
Landmarks used on cranium for 3D geometric morphometrics. (**A**) Dorsal view of cranium showing position of landmarks 1–19, (**B**) Lateral view of cranium showing position of landmarks 20–23. Anatomical landmark descriptions defined in Section 2. Materials and Methods.

The geometric morphometric analysis for the 3D models was performed using MorphoJ v1.07a [29]. All landmarks were applied to models and images twice to test for digitizing error [30]. Landmarks used were chosen from similar studies of small mammalian craniums [31,32,33]. The digitizing error was rendered to non-significant using revision where required. The cranium landmarks used were (1) anteromedial point on the nasal, (2) midsagittal point of the frontonasal suture, 3 (R) and 4 (L) most dorsal point of the nasal (right and left), 5 (R) and 6 (L) intersection of the frontal, maxilla, and lacrimal suture (right and left), 7 (R) and 8 (L) intersection of maxilla, nasal and temporal suture (right and left), 9 (R) and 10 (L) anterior intersection of the nasal and maxilla suture (right and left), 11 (R) and 12 (L) most posterior point on the nasal (right and left), 13 (R) and 14 (L) post-orbital process of the frontal (right and left), (15) intersection of frontal and parietal suture, (16) intersection of parietal and occipital suture, (17) posterior most point on the sagittal crest, 18 (L) and 19 (R) posterior point on temporal along zygomatic process, (20) medial point on the dorsal border of the foramen magnum, (21) anteromedial most point on the incisive and 22 (L) and 23 (R) posterior border of the C1 alveolus. Analysis performed for geometric morphometrics included discriminant function analysis (DFA), canonical variates analysis (CVA), and principal component analysis (PCA) (Figure 2).

**Figure 3:**
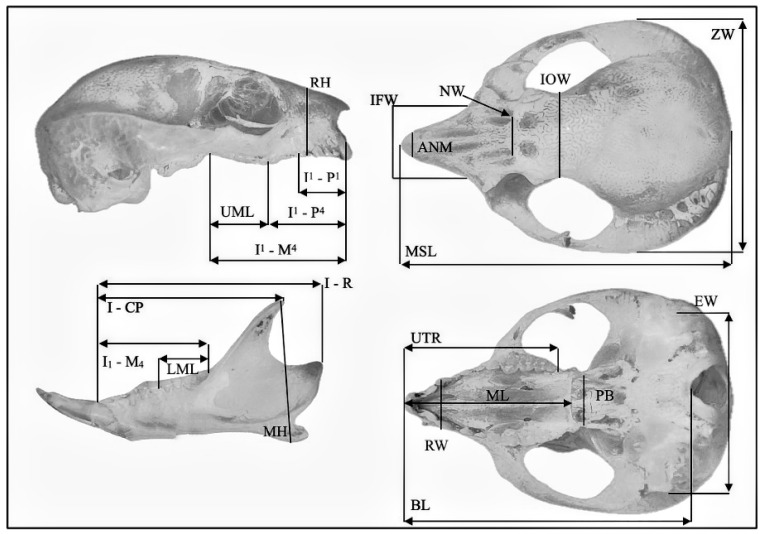
Linear measurements of the cranium and abbreviations. Linear measurements defined in Section 2. Materials and Methods).

Measurements of the cranium were collected by the primary author using Protech Digital Vernier Callipers. The linear measurements chosen were based on studies with similar sized mammalian species [9,34,35]. The craniodental features measured included the following: MSL = maximum skull length; BL = basicranial skull length including incisors; ZM = maximum width of cranium on zygomatic bones; IOW = width if interorbital constriction where sutures meet the orbital cavities; IFW = width between the infraorbital foramen; NW = width of nasal bones at the nasal/premaxilla/maxilla junction; ANM = width in nasal bones at nasal bones and maxilla intersection, RH = rostral height at canine to point over the rostrum; RW = width of rostrum at the highest point of the canines on the maxilla; I^1^-P^4^ = insertion point of upper incisor 1 to upper premolar 4; I^1^-P^1^ = insertion point of upper incisor 1 to upper premolar 1; UTR = full length upper tooth row including front incisor; UML = upper molar length; ML = maxilla length; PB = width of palatine region; M^1^-M^4^; EW = length between lateral openings to auditory process; MH = maximum height of the ramus; LML = lower molar length M_1_-M_4_; I-CP = length from insertion point of I_1_ to coronal process; I-R = length from insertion point of I_1_ to distal ramus; I_2_-M_4_ = length from insertion point I_2_ to M_4_ (Figure 2). Comparisons of size of the craniums were analyzed using discriminant function analysis (DFA), canonical variates analysis (CVA) and ANOVA using JMP^®^ Version 16.2.0. (SAS Institute Inc., Cary, NC, USA, 1989–2023) (Figure 3).

Sexual dimorphism using DFA with 1000 permutations was examined for the shape of the craniums. No shape characteristics of sexual dimorphism was identified for the cranium for *P. breviceps*, *P. notatus* and *P. papuanus* (Supplementary Materials). All subsequent analyses of shape used combined male and female populations for each group. The size characteristics of sexual dimorphism identified between males and females for skull size and cranium size were found (Supplementary Materials). Subsequent size analysis used separated male and female populations for each region. Effects of allometry (size on shape) was assessed by a regression analysis in MorphoJ. The Procrustes coordinates and centroid size were compared to quantify the amount of shape variation influenced as the size of the specimen increased. Less than 1.5% of the shape change was explained by the size increase, and the permutation test against the null hypothesis of independence was not significant (Supplementary Materials).

## 3. Results

**Figure 4:**
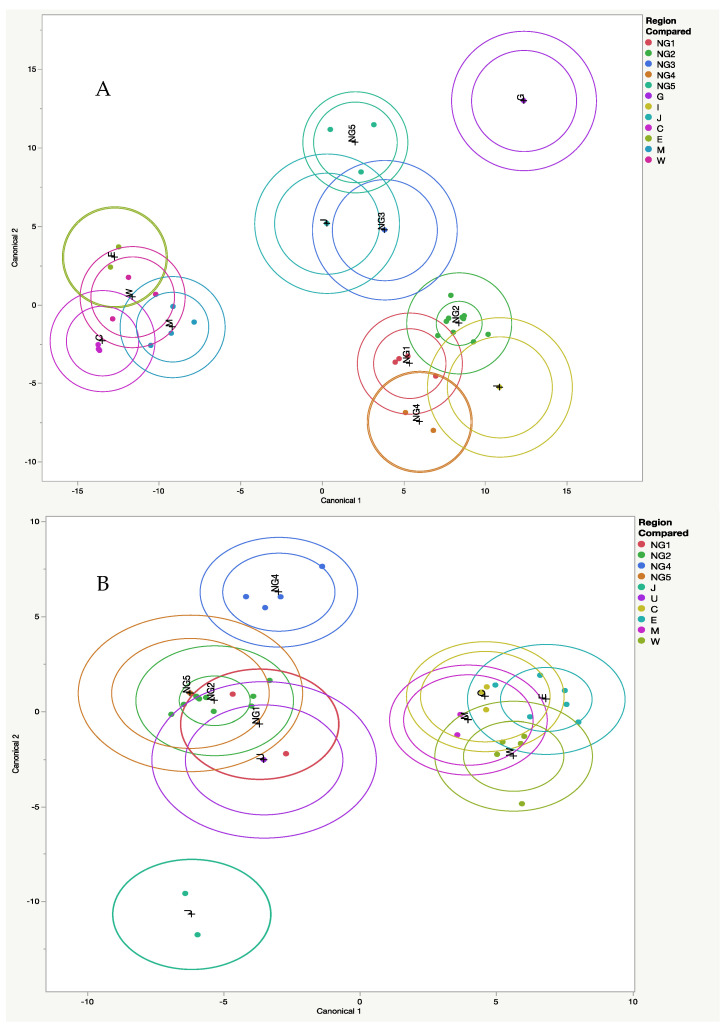
Canonical VARIATES ANALYSIS for size for all regions compared in Queensland and New Guinea. (**A**) Male specimens compared. (**B**) Female specimens compared. NG1, NG2, NG3, NG4, NG5, and B (New Britain Island), G (Goodenough Island), I (Misima Island), J (Irian Jaya), S (Solomon Islands), and U (southeast corner Papua New Guinea). Australian specimens were allocated to C (Cape York region), E (east coast Queensland), M (region of Great Dividing Range (GDR)) and W (West of GDR). (NG clades defined in Section 2. Materials and Methods).

Significant size differences were found among Queensland and New Guinea. Where insufficient numbers of specimens were available, regions for males or females were not compared (Table 1). Canonical variates analysis of the compared groups showed a clustering for both males and females and between Queensland and New Guinea (Figure 4). There was a significant different found when all regions were compared for males (Wilks’ Λ = 1.0789 × 10^−8^, F(66.6, 231) = 1.59, *p* = 0.01) and females (Wilks’ Λ = 4.6131 × 10^−6^, F(76.9, 189) = 1.37, *p* = 0.05). Regions of the skull that were compared indicate that for males, UML (upper molar length), EW (length between lateral openings to auditory processes), and I^1^-P^1^ (insertion point of upper incisor 1 to upper premolar 1) were significantly different for size across all compared regions. For females, ZW (zygomatic width), I^1^-P^1^, EW, UML, ICP (length from insertion point of I_1_ to coronal process) and I-R (length from insertion point of I_1_ to distal ramus) were significantly different in size (Supplementary Materials).

**Figure 5:**
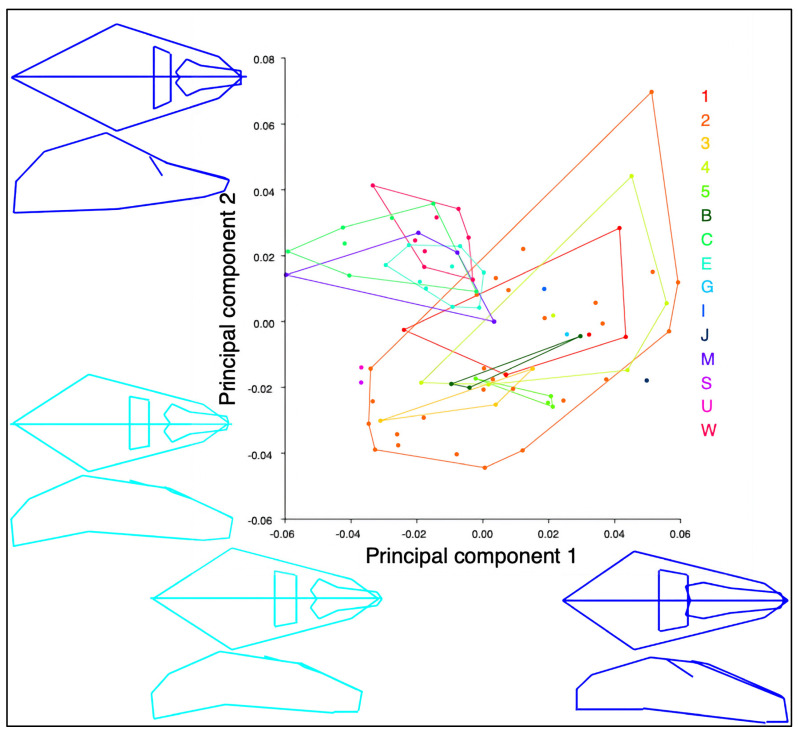
Principal component analysis for all Queensland and New Guinea specimens. PC1 (23% of variation) on *X*-axis and PC2 (16% of variation) on *Y*-axis. Light blue and dark blue wireframes represent shape change observed (below *X*-axis PC1 and left of *Y*-axis PC2). Convex hulls used for each group of specimens. Regions in analysis include 1 (NG1), 2 (NG2), 3 (NG3), 4 (NG4), 5 (NG5), and B (New Britain Island), G (Goodenough Island), I (Misima Island), J (Irian Jaya), S (Solomon Islands), and U (southeast corner Papua New Guinea). Australian specimens were allocated to C (Cape York region), E (east coast Queensland), M (region of Great Dividing Range (GDR)) and W (west of GDR) (NG clades defined in Section 2. Materials and Methods).

**Figure 6:**
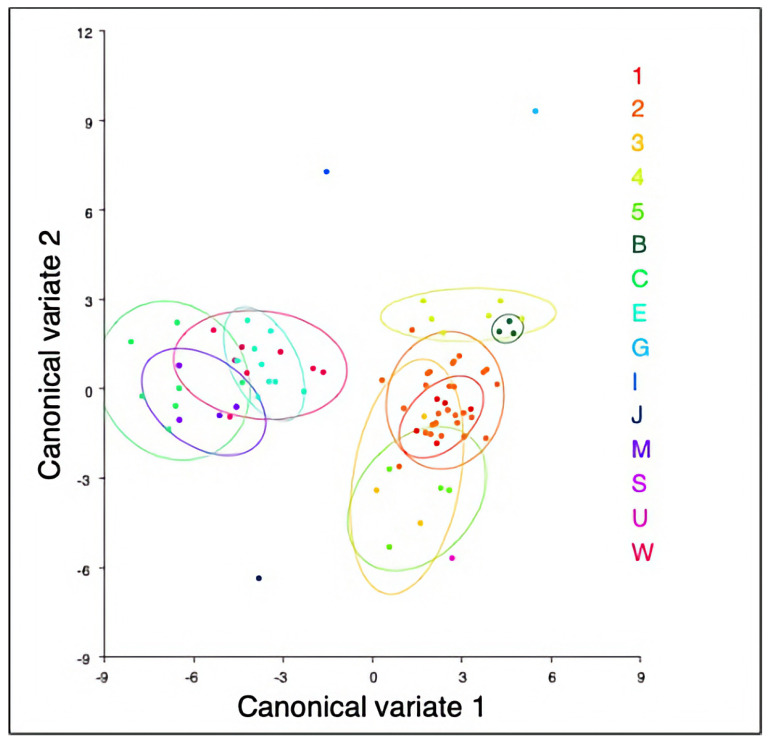
Canonical variates analysis for shape of Australian and New Guinea regions. Right cluster is New Guinea regions and left cluster is Australian regions. New Guinea specimens allocated to 1 (NG1), 2 (NG2), 3 (NG3), 4 (NG4), 5 (NG5), and B (New Britain Island), G (Goodenough Island), I (Misima Island), J (Irian Jaya), S (Solomon Islands), and U (southeast corner Papua New Guinea). Australian specimens were allocated to C (Cape York region), E (east coast Queensland), M (region of Great Dividing Range (GDR)) and W (west of GDR). NG clades defined in Section 2. Materials and Methods.

Principal component analysis showed that over 75% of the variation was explained by the first eight PCs. PC1 showed a wider to narrowing brain case and shorter to longer rostrum. PC2 showed a wider to narrowing of the cranium at the zygomatics and lengthening of the rostrum. Using convex hulls, the Australian and New Guinea specimens clustered separately (Figure 5 and Supplementary Materials). Using canonical variates analysis, two clusters of specimens were found from Australia and New Guinea (Figure 6).

Significant shape changes were found between Australian and New Guinea groups of specimens. Specimens located in Cape York were significantly different to all identified NG clades identified in Malekian et al. (2010) [10]. The Cape York specimens were significantly different from the eastern Queensland specimens, but they had no significant shape change from the middle or western species. No significant shape change was found between the east, middle or western Queensland specimens. The east, mid and west regions of Queensland had significant differences in shape from several of the New Guinea regional species. Between the New Guinea regions, NG5 was significantly different from NG2, NG4, Goodenough Island and Irian Jaya. NG2 was significantly different from New Britain Island. No other significant shape change was found in the New Guinea regions (Table 1 and Supplementary Materials).

## 4. Discussion

**Figure 7:**
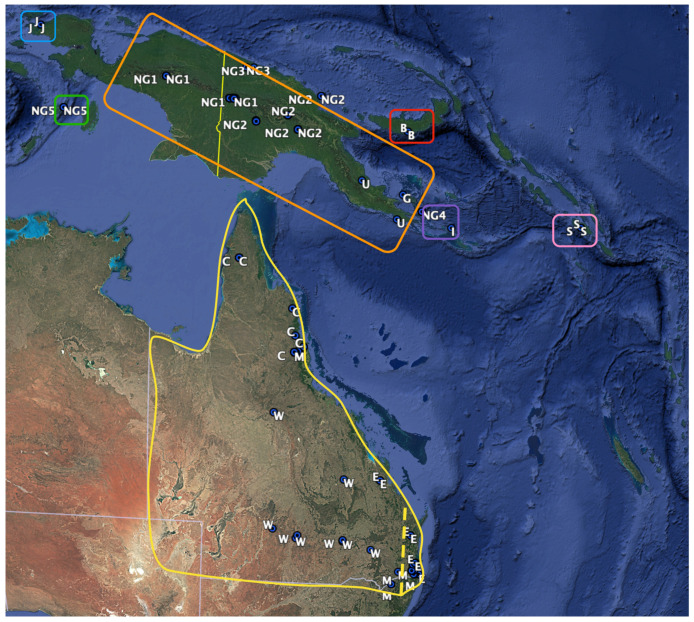
Revised distribution for *P. notatus and P. papuanus*. Northeastern Australia *P. notatus* (yellow border), dotted line showing residual *P. breviceps* population. New Guinea mainland, *P. papuanus* (orange border) no significant difference in shape between previously identified clades. Solomon Islands (pink), New Britain (red), Kai Island (green), Normanby Island (purple) and Irian Jaya (blue) indicate significant shape variation from mainland populations. (Image developed using Google Earth Pro (2022) 7.3.6.9345 (64-bit) Data SIO, NOAA, US. Navy, NGA, GEBCO Image Landsat/Copernicus).

The review of the taxonomy of the *Petaurus* genus and particularly *P. breviceps* for this research has found evidence to consolidate the identification and distribution for the species. The widely distributed *P. notatus* throughout Australia likely extends to the Cape York region in the northeast of the mainland. Evidence supports the limit of their distribution as the TransFly region of New Guinea, which is a sea region with multiple islands separated by small distances between Australia and New Guinea [2]. Mainland New Guinea *P. papuanus* revealed significantly different sized skulls, but shape differences were not found. In the surrounding islands of New Guinea, morphological variation was identified, indicating possible subspecies (Figure 7).

The *Petaurus* genus in Australia’s northeastern mainland is likely the result of at least one dispersal event from New Guinea to Australia [10]. The early identification of labeled *P. breviceps* in Cape York and throughout north Queensland was of *P. b. longicaudatus* (now recognized as *P. notatus*), and the results from this research support this interpretation. The Cape York cranium shape was not significantly different when compared with known *P. notatus* craniums. No significant shape changes for the cranium were found for specimens from Cape York and the west or the middle GDR regions, which were the described geographic regions for this species [9]. Furthermore, they were significantly different from species located throughout New Guinea (*P. papuanus*) and southeastern Queensland labeled *P. breviceps*. There were significant size differences found between all compared regions of Queensland. Previous evidence for significant size differences between populations of *Petaurus* sp. in Australia has been reported, which was likely a result of the climatic effect of Bergmann’s Rule [36]. Specimens in Cape York are likely affected by this climatic principle and have a reduced body size suitable for the tropical conditions found there. Currently labeled Australian *P. breviceps* from the Cape York region in northeast Queensland should be recognized as *P. notatus*.

Specimens in New Guinea were significantly different in shape compared to Australian regions. No evidence was found to support the continuation of a species phenotype throughout Australia and New Guinea, which was subsequent to the divergence event between ~3.5 and 8.4 mya [10]. Whether the resultant *P. papuanus* in New Guinea or *P. notatus* in Queensland was the founding species is unclear. There was no evidence for any northern Australian or New Guinea cranium shape similarity with *P. breviceps*, which is a species recognized in a narrow channel in coastal mid-eastern Australia. *P. breviceps* should be considered a divergent species from *P. notatus* due to their isolated geographic distribution.

The significant shape differences found in New Guinea between NG5 (Kai Island) and NG2 throughout the New Guinea central mainland and the northwest Irian Jaya (previously unsampled), as well as NG4 in the central–east island (Normanby Island) and Goodenough Island, also a central eastern island, reveal diversity in skull shape in these island populations. Significant size differences were also found between NG2 and New Britain Island in the northeast of New Guinea. The isolation of these island populations of *P. papuanus* have likely contributed to the evolution of the morphological shape of the cranium and the variation found. Research suggests that the evolution rate of island mammals is accelerated when compared with mainland populations [37]. Mammals respond to their novel environment quickly, and this is associated with a faster rate of morphological variation and changes in size when compared with mainland conspecifics [38,39]. The size variation found among the species in New Guinea, and the morphological shape variation found among island populations is consistent with previous research for island populations. No significant shape changes were found among the three NG clades on mainland New Guinea. The New Guinea mainland identified clades are likely co-located throughout their mainland distributions. Previously reported introgression may be reflected in these results as no significant shape changes in the cranium of New Guinea mainland clades was seen [40]. Other ecological forces that might affect the shape of a cranium, such as diet, may also be similar throughout the New Guinea mainland distribution, rendering dietary specialization and associated morphological differences unlikely. Hybridization events, similarity in habitat and diet found throughout the tropical locations of New Guinea may cause the identification of morphological characteristics to define each clade with their cranium challenging.

Our research has used museum specimens for 3D scans and linear measurements of the cranium for the Queensland and New Guinea specimens. A limitation of this research may be in the number of specimens available for this analysis. For some regions, particularly in New Guinea, there was a lack of both sexes in the museum collection, and specimens were available from a single time period.

## 5. Conclusions

To contribute to the resolution of taxonomy for the northern Queensland labeled *P. breviceps* and New Guinea labeled *P. papuanus*, we compared the cranium of specimens for shape and size variation between known *P. notatus* and *P. breviceps* species. No significant shape difference was found between the Cape York and *P. notatus* specimens in northeast Australia. Our research supports the identification of *P. notatus* here. All compared specimens of *P. notatus* throughout Queensland were significantly different in size, with the Cape York specimens having the smallest skull size, which was consistent with a climatic response found in other *Petaurus* sp. in Australia. There were significant shape and size changes found between Queensland and the New Guinea regions. Our research has found significant shape changes between New Guinea islands and mainland groups. Size variation between most groups was also found. There is evidence to support island evolution for morphology and size as isolated from mainland New Guinea specimens. No significant shape difference was found in the mainland New Guinea compared clades from Malekian et al. (2010) [10], which was likely due to shared distribution and hybridization events.

## Figures and Tables

**Table 1:** Results for shape and size DFA between regions in Queensland and New Guinea. Top right corner of figure shows compared regions for DFA of shape; red indicated significant shape change between regions. Bottom left corner shows DFA for size between regions. Green shows significant size differences (males and females), pink show significant size difference for females where there were insufficient males to compare, blue shows significant size difference for males where there were insufficient females to compare. ‘Nt’ was not tested due to insufficient number of specimens. White squares show no significant difference.

	NG1	NG2	NG3	NG4	NG5	B	G	I	J	S	U	C	E	M	W
NG1															
NG2															
NG3															
NG4															
NG5															
B	Nt	Nt	Nt	Nt	Nt										
G			Nt			Nt		Nt	Nt	Nt	Nt				
I			Nt			Nt	Nt		Nt	Nt	Nt				
J			Nt			Nt	Nt	Nt		Nt	Nt				
S	Nt	Nt	Nt	Nt	Nt	Nt	Nt	Nt	Nt		Nt				
U	Nt		Nt		Nt	Nt	Nt	Nt		Nt					
C						Nt	Nt	Nt		Nt					
E						Nt	Nt	Nt		Nt					
M						Nt	Nt	Nt		Nt					
W						Nt	Nt	Nt		Nt					

## Data Availability

The datasets presented in this article are not readily available because the data are part of an ongoing study. Requests to access the datasets should be directed to mpowley@uow.edu.au.

## Supplementary Materials

The following supporting information can be downloaded at https://www.mdpi.com/article/10.3390/ani14243680/s1; Table S1: Results from DFA comparing size for all regions in New Guinea and Australia. Order of results for shape (Top right corner (gray) Procrustes distance, Mahalanobis distance, T-square, *p*-value (parametric), *p*-values for permutation tests (10,000 permutation runs): Procrustes distance, T-square. Bottom left corner (white) *p* values for significant size differences. For full description of region abbreviations, see Section 2. Materials and Methods. Table S2: Female mean sizes for each linear measurement taken of the skull for each region (mm). For full description of abbreviations see Section 2. Materials and Methods. Table S3: Male mean sizes for each linear measurement taken of the skull for each region (mm). For full description of abbreviations Section 2. Materials and Methods. Table S4: PCA results for shape of cranium with variance and cumulative values to 100%.
