# Supplementary material for: Comparative Analysis of Petaurus Cryptic Species of ‘Sugar Glider’ from Australia and New Guinea Using 3D Geometric Morphometrics
Source: Animals (Basel). 2024 Dec 20;14(24):3680. doi: 10.3390/ani14243680 (PMC11672710; doi:10.3390/ani14243680)
Supplement: Supplementary file 1 [file animals-14-03680-s001.zip › animals-3334808-supplementary.pdf]

Table S1: Results from DFA comparing size for all regions in New Guinea and Australia. Order of results for shape (Top right corner (grey) Procrustes distance, Mahalanobis distance, T-square, P-value (parametric), P-values for permutation tests (10000 permutation runs): Procrustes distance, T-square. Bottom left corner (white) P values for significant size differences. For full description of region abbreviations see Materials and Methods.

	NG1	NG2	NG3	NG4	NG5	B	G	I	J	S	U	C	E	M	W
**NG1**		0.03552446 10.0171 427.9266, 0.8683 (10000 permutation runs): 0.1283 0.3109	0.04889925 1.7355 4.3029, 0.9392 (10000 permutation runs): 0.3778 0.2395	0.04002217 2.1911 13.0931, 0.9654 (10000 permutation runs): 0.2542 0.1657	0.03314107 0.9560 2.0311, 0.9983 (10000 permutation runs): 0.5547 0.6764	0.05228057 1.5300 4.3894, 0.9713 (10000 permutation runs): 0.0738 0.1895	0.07396477 1.5207 1.9270, 0.9551 (10000 permutation runs): 0.2391 0.4997	0.04446543 0.4565 0.1737, 0.9993 (10000 permutation runs): 0.8403 0.8529	0.07426498 2.6497 5.8510, 0.8265 (10000 permutation runs): 0.4890 0.4711	0.10131293 2.4637 5.0580, 0.8501 (10000 permutation runs): 0.0704 0.1753	0.08346893 1.8734 2.9248, 0.9205 (10000 permutation runs): 0.1825 0.3304	0.06636804 4.4066 56.6374, 0.7866 (10000 permutation runs): 0.0008 0.0020	0.04488025 5.2603 88.9401, 0.7727 (10000 permutation runs): 0.0035 0.0328	0.05518769 2.1572 10.3410, 0.9340 (10000 permutation runs): 0.0492 0.0238	0.05286819 3.7171 42.5137, 0.8803 (10000 permutation runs): 0.0008 0.0005
**NG2**	**M<0.0001 F<0.0001**		0.04141370 10.0280 188.1458, 0.9568 (10000 permutation runs): 0.4001 0.5572	0.03604106 7.3780 270.6220, 0.9469 (10000 permutation runs): 0.0791 0.9349	0.02893308 23.3663 1919.2216, 0.5155 (10000 permutation runs): 0.5052 0.0178	0.03829668 28.4150 2195.1542, 0.4732 (10000 permutation runs): 0.2908 0.0030	0.06661780 18.5746 333.5160, 0.8636 (10000 permutation runs): 0.2570 0.0998	0. 0.04580420 17.7323 303.9536, 0.8805 (10000 permutation runs): 0.7905 0.2998	0.07829126 16.0584 249.2756, 0.9130 (10000 permutation runs): 0.0801 0.2225	0.08217480 18.9825 348.3259, 0.8553 (10000 permutation runs): 0.0973 0.1354	0.06563468 12.2925 146.0677, 0.9719 (10000 permutation runs): 0.2710 0.4460	0.05674857 Mahalanobis distance: 235.7832 313486.7549, 0.0481 (10000 permutation runs): <.0001 0.0449	0.03974376 36.7066 9254.3169, 0.0125 (10000 permutation runs): 0.0018 0.0122	0.04678169 15.6734 863.5119, 0.7004 (10000 permutation runs): 0.0337 0.0034	0.04932929 26.5294 4413.0587, 0.1257 (10000 permutation runs): <.0001 0.1281
**NG3**	**M<0.0001** F=nt	**M<0.0001** F=nt		0.05013218 0.9890 1.4673, 0.9974 (10000 permutation runs): 0.4064 0.6880	0.04243541 0.9530 1.2110, 0.9779 (10000 permutation runs): 0.2246 0.0965	0.05039677 0.9521 1.0878, 0.9363 (10000 permutation runs): 0.1707 0.2704	0.08330216 0.4591 0.1405, 0.7717 (10000 permutation runs): 0.3346 0.6689	0.06324025 0.2611 0.0455, 0.8663 (10000 permutation runs): 0.6671 0.3307	0.06232028 0.3963 0.1047, 0.8008 (10000 permutation runs): 0.6636 0.3360	0.09898676 0.2333 0.0363, 0.8802 (10000 permutation runs): 0.3319 1.0000	0.08630170 0.0381 0.0010, 0.9802 (10000 permutation runs): 0.3275 1.0000	0.07000441 3.5455 19.5541, 0.8426 (10000 permutation runs): 0.0006 0.0013	0.05537836 3.7140 22.5711, 0.9093 (10000 permutation runs): 0.0283 0.1477	0.06232028 0.3963 0.1047, 0.8008 (10000 permutation runs): 0.6636 0.3360	0.05959871 3.9014 24.3538, 0.8563 (10000 permutation runs): 0.0167 0.0123
**NG4**	**M<0.0001**	**M<0.0001**	**M<0.0001**		0.04625540	0.04161898	0.05183514	0.05529787	0.08308004	0.08916730	0.08074897	0.07004578	0.05398708	0.05963479	0.06045093

	NG1	NG2	NG3	NG4	NG5	B	G	I	J	S	U	C	E	M	W
	**F<0.0001**	**F<0.0001**	F=nt		2.8190<br>19.0720, 0.8957 (10000 permutation runs): 0.0799<br>0.0337	1.4912<br>4.4476, 0.9872 (10000 permutation runs): 0.4504<br>0.3513	0.3689<br>0.1166, 1.0000 (10000 permutation runs): 0.6596<br>0.8767	1.8728<br>3.0064, 0.9656 (10000 permutation runs): 0.5868<br>0.3213	1.5988<br>2.1911, 0.9803 (10000 permutation runs): 0.1909<br>0.3486	2.2383<br>4.2943, 0.9393 (10000 permutation runs): 0.2726<br>0.2184	2.5954<br>5.7738, 0.9080 (10000 permutation runs): 0.2016<br>0.2033	4.2482<br>58.3054, 0.8224 (10000 permutation runs): 0.0005<br>0.0015	4.8329<br>84.0865, 0.8202 (10000 permutation runs): <.0001<br>0.0014	3.3774<br>27.3762, 0.8352 (10000 permutation runs): 0.0102<br>0.0027	4.8122<br>79.3959, 0.7970 (10000 permutation runs): <.0001<br>0.0005
**NG5**	**M<0.0001**<br>**F<0.0001**	**M<0.0001**<br>**F<0.0001**	**M<0.0001**<br>F=nt	**M<0.0001**<br>**F<0.0001**		0.05309297<br>2.5907<br>11.5059, 0.7995 (10000 permutation runs): 0.0594<br>0.0771	0.07816926<br>2.2458<br>4.0351, 0.7678 (10000 permutation runs): 0.0150<br>0.0482	0.05780708<br>0.4813<br>0.1853, 0.9939 (10000 permutation runs): 0.2005<br>0.1022	0.07085543<br>1.2732<br>1.2968, 0.9220 (10000 permutation runs): 0.1970<br>0.0504	0.10065852<br>2.8381<br>6.4437, 0.6776 (10000 permutation runs): 0.0677<br>0.1669	0.07817279<br>2.5335<br>5.1348, 0.7226 (10000 permutation runs): 0.1137<br>0.0881	0.07100866<br>3.4583<br>30.4427, 0.8627 (10000 permutation runs): 0.0002<br>0.0007	0.05229307<br>3.1321<br>27.1667, 0.9415 (10000 permutation runs): 0.0007<br>0.0750	0.06054477<br>2.4733<br>12.2348, 0.8629 (10000 permutation runs): 0.0082<br>0.0098	0.06384879<br>3.5927<br>34.4199, 0.8809 (10000 permutation runs): 0.0022<br>0.0014
**B**	M=nt<br>F=nt	M=nt<br>F=nt	M=nt<br>F=nt	M=nt<br>F=nt	M=nt<br>F=nt		0.05579669<br>0.7532<br>0.4255, 0.9081 (10000 permutation runs): 0.4203<br>0.7490	0.06403864<br>0.9131<br>0.6254, 0.8728 (10000 permutation runs): 0.2571<br>0.6610	0.09359960<br>1.3304<br>1.3275, 0.7753 (10000 permutation runs): 0.2513<br>0.1665	0.07244971<br>1.0243<br>0.7869, 0.8471 (10000 permutation runs): 0.5012<br>0.3343	0.07063528<br>1.2333<br>1.1408, 0.7980 (10000 permutation runs): 0.5042<br>0.3405	0.06831247<br>2.4100<br>12.1970, 0.9488 (10000 permutation runs): 0.0018<br>0.0412	0.05262720<br>3.2897<br>24.3505, 0.9298 (10000 permutation runs): 0.0021<br>0.1293	0.06455258<br>2.8652<br>14.0733, 0.7599 (10000 permutation runs): 0.0076<br>0.0272	0.06368350<br>4.5137<br>44.4507, 0.7900 (10000 permutation runs): 0.0055<br>0.0046
**G**	**M<0.0001**	**M<0.0001**	M=nt	**M<0.0001**	**M<0.0001**	M=nt		Nt	Nt	Nt	Nt			0.117	**0.014**

	NG1	NG2	NG3	NG4	NG5	B	G	I	J	S	U	C	E	M	W
	F=nt	F=nt	F=nt	F=nt	F=nt	F=nt						0.09375433<br><br>3.5664<br><br>11.1291, 0.8778 (10000 permutation runs): 0.0061 0.0764	0.07682231<br><br>3.3295<br><br>9.9769, 0.9649 (10000 permutation runs): 0.1447 0.2662		
**I**	**M<0.0001**<br>F=nt	**M<0.0001**<br>F=nt	M=nt<br>F=nt	**M<0.0001**<br>F=nt	**M<0.0001**<br>F=nt	M=nt<br>F=nt	M=nt<br>F=nt		Nt	Nt	Nt	0.06990770<br><br>1.7859<br><br>2.7907, 0.9885 (10000 permutation runs): 0.0032 0.4620	0.05457396<br><br>2.8620<br><br>7.3720, 0.9815 (10000 permutation runs): 0.2782 0.4166	0.06010206<br><br>1.4370<br><br>1.6520, 0.8982 (10000 permutation runs): 0.2985 0.2236	0.767
**J**	**M<0.0001**	**M<0.0001**	M=nt	**M<0.0001**	**M<0.0001**	M=nt	M=nt	M=nt		Nt	Nt				**0.018**

	**NG1**	**NG2**	**NG3**	**NG4**	**NG5**	**B**	**G**	**I**	**J**	**S**	**U**	**C**	**E**	**M**	**W**
	**F<0.0001**	**F<0.0001**	F=nt	**F<0.0001**	**F<0.0001**	F=nt	F=nt	F=nt				0.10773263<br><br>4.4388<br><br>17.2402, 0.8014 (10000 permutation runs): 0.0005 0.0293	0.09151189<br><br>7.7122<br><br>53.5305, 0.6940 (10000 permutation runs): 0.0807 0.0590	0.09397401<br><br>2.2040<br><br>3.8861, 0.7746 (10000 permutation runs): 0.0523 0.0474	
**S**	M=nt F=nt	M=nt F=nt	M=nt F=nt	M=nt F=nt	M=nt F=nt	M=nt F=nt	M=nt F=nt	M=nt F=nt	M=nt F=nt		Nt	Difference between means: 0.08463735<br><br>2.8233<br><br>6.9744, 0.9365 (10000 permutation runs): 0.0382 0.0945	0.09043455<br><br>6.4827<br><br>37.8227, 0.7705 (10000 permutation runs): 0.0120 0.1722	0.08790433<br><br>1.5381<br><br>1.8925, 0.8827 (1000 permutation runs): 0.0330 0.0790	0.09621374<br><br>4.3674<br><br>16.9546, 0.8671 (1000 permutation runs): 0.0670 0.0680
**U**	M=nt	M=nt	M=nt	M=nt	M=nt	M=nt	M=nt	M=nt	M=nt	M=nt					

	NG1	NG2	NG3	NG4	NG5	B	G	I	J	S	U	C	E	M	W
	F ns	**F<0.0001**	F=nt	**F<0.0001**	F=nt	F=nt	F=nt	F=nt	**F<0.0001**	F=nt		0.06990956<br><br>1.8963<br><br>3.1465, 0.9852 (10000 permutation runs): 0.0756 0.3929	0.06429168<br><br>3.1512<br><br>8.9370, 0.9719 (10000 permutation runs): 0.1232 0.3571	0.07314843<br><br>1.4451<br><br>1.6706, 0.8970 (1000 permutation runs): 0.1690 0.4560	0.07751582<br><br>2.6882<br><br>6.4234, 0.9720 (1000 permutation runs): 0.0310 0.0160
**C**	**M<0.0001**	**M<0.0001**	**M<0.0001**	**M<0.0001**	**M<0.0001**	M=nt	M=nt	M=nt	**M<0.0001**	M=nt	M=nt		0.03475833<br><br>4.4108<br><br>76.6038, 0.8680 (10000 permutation runs): 0.0047 0.0022	0.02741426<br><br>2.0071<br><br>10.2545, 0.9796 (10000 permutation runs): 0.4314 0.4440	0.03172864<br><br>2.0602<br><br>15.8462, 0.9939 (10000 permutation runs): 0.0183 0.1255
	**F<0.0001**	**F<0.0001**	F=nt	**F<0.0001**	**F<0.0001**	F=nt	F=nt	F=nt	**F<0.0001**	F=nt	**F<0.0001**				
**E**	**M<0.0001**	**M<0.0001**	**M<0.0001**	**M<0.0001**	**M<0.0001**	M=nt	M=nt	M=nt	**M<0.0001**	M=nt	M=nt	**M<0.0001**			

	NG1	NG2	NG3	NG4	NG5	B	G	I	J	S	U	C	E	M	W
	**F<0.0001**	**F<0.0001**	F=nt	**F<0.0001**	**F<0.0001**	F=nt	F=nt	F=nt	**F<0.0001**	F=nt	**F<0.0001**	**F<0.0001**		0.0300927<br>4<br><br>3.0735<br><br>26.1588, 0.9454 (10000 permutation runs): 0.2744 0.0933	0.0276432<br>7<br><br>3.1123<br><br>41.0238, 0.9666 (10000 permutation runs): 0.0366 0.0866
**M**	**M<0.0001**<br>**F<0.0001**	**M<0.0001**<br>**F<0.0001**	**M<0.0001**<br>F=nt	**M<0.0001**<br>**F<0.0001**	**M<0.0001**<br>**F<0.0001**	M=nt<br>F=nt	M=nt<br>F=nt	M=nt<br>F=nt	**M<0.0001**<br>**F<0.0001**	M=nt<br>F=nt	M=nt<br>**F<0.0001**	**M<0.0001**<br>**F<0.0001**	**M<0.0001**<br>**F<0.0001**		0.0255314<br>1<br><br>1.3341<br><br>4.7463, 0.9990 (1000 permutation runs): 0.5710 0.8880
**W**	**M<0.0001**<br>**F<0.0001**	**M<0.0001**<br>**F<0.0001**	**M<0.0001**<br>F=nt	**M<0.0001**<br>**F<0.0001**	**M<0.0001**<br>**F<0.0001**	M=nt<br>F=nt	M=nt<br>F=nt	M=nt<br>F=nt	**M<0.0001**<br>**F<0.0001**	M=nt<br>F=nt	M=nt<br>**F<0.0001**	**M<0.0001**<br>**F<0.0001**	**M<0.0001**<br>**F<0.0001**	**M<0.0001**<br>**F<0.0001**	

Table S2: Female mean sizes for each linear measurement taken of the skull for each region (mm). For full description of abbreviations see Materials and Methods.

Female	NG1	NG2	NG4	NG5	J	U	C	E	M	W
**MSL**	35.68	35.05	37.78	36.36	34.03	35.68	34.81	36.82	35.36	35.67
**BL**	31.72	30.79	33.79	32.86	30.32	31.93	31.16	32.47	30.68	31.28
**ZW**	23.91	23.64	24.60	24.55	24.26	24.65	23.90	25.48	24.05	25.77
**IOW**	7.30	7.31	7.87	7.06	7.04	7.22	7.03	7.45	7.22	7.71
**LW**	7.89	7.97	8.87	8.34	7.68	8.24	8.05	8.44	8.04	8.54
**NW**	6.32	6.13	5.64	6.48	7.05	6.26	6.01	6.47	6.51	6.21
**ANM**	2.66	2.64	2.89	2.93	2.81	2.81	2.29	2.64	2.45	2.77
**RH**	8.55	8.40	9.43	8.89	8.34	9.05	8.18	8.58	8.26	8.46
**ROW**	5.93	6.21	6.21	6.55	5.73	6.23	6.51	6.26	6.63	6.57
**UTR**	16.41	16.46	17.52	16.31	16.00	16.89	16.58	17.44	16.79	16.74
**UML**	5.66	6.18	6.65	6.09	5.51	6.38	6.60	6.61	6.64	6.69
**I1-P4**	11.11	10.65	11.61	11.45	9.47	10.69	10.65	11.48	10.92	10.79
**I1-P1**	7.92	7.41	8.39	8.02	7.33	7.84	8.53	9.52	8.80	9.21
**EW**	19.92	19.57	19.76	20.09	20.57	21.50	20.68	22.46	21.03	22.50
**PB**	6.55	6.57	6.50	6.28	6.70	6.37	6.62	6.88	6.41	6.67
**ML**	19.13	18.49	20.00	19.82	18.19	18.41	17.62	18.87	17.99	18.04
**LML**	7.08	7.01	7.86	7.85	7.46	7.22	7.44	7.41	7.49	7.30
**MR**	11.76	11.70	12.79	12.23	11.94	12.84	11.63	12.95	12.00	12.93
**RL1**	19.88	19.08	21.30	20.69	18.28	20.44	17.58	19.41	18.28	18.47
**RL2**	21.67	21.16	23.16	22.79	20.33	22.36	20.49	22.18	21.44	21.21
**I2-M4**	10.60	10.45	11.26	11.18	10.26	10.63	10.37	10.81	10.48	10.51

Table S3: Male mean sizes for each linear measurement taken of the skull for each region (mm). For full description of abbreviations see Materials and Methods.

Male	NG1	NG2	NG3	NG4	NG5	G	I	J	C	E	M	W
**MSL**	35.72	36.19	38.85	36.83	37.93	38.84	33.69	34.93	34.86	37.04	37.18	37.28
**BL**	31.29	31.87	34.23	33.17	33.61	35.23	29.32	31.04	30.55	32.78	32.49	32.87
**ZW**	24.72	24.51	25.10	24.69	26.37	23.35	23.22	24.54	24.28	25.62	26.40	26.11
**IOW**	7.45	7.44	7.78	7.60	7.05	6.89	7.21	7.19	7.02	7.40	7.80	7.31
**LW**	8.28	8.44	8.76	8.74	8.54	7.97	8.07	8.19	8.22	8.46	8.49	8.56
**NW**	6.02	6.18	6.61	6.12	6.43	5.84	5.10	6.01	6.45	6.77	6.75	6.45
**ANM**	2.91	2.98	2.99	3.14	2.85	2.69	2.82	2.51	2.58	2.63	2.76	2.63
**RH**	8.67	8.82	9.07	9.35	9.78	8.85	7.85	8.49	8.23	8.96	8.95	8.95
**ROW**	6.55	6.42	6.48	7.03	6.57	6.50	5.91	6.45	6.64	6.59	6.78	6.60
**UTR**	16.17	16.85	17.75	17.25	17.13	18.61	14.84	15.80	16.53	17.54	17.53	17.85
**UML**	5.72	6.15	5.79	5.96	6.50	7.35	5.80	6.58	6.83	6.94	6.81	7.00
**I1-P4**	10.97	11.08	11.90	11.28	11.17	12.34	10.51	10.38	11.17	11.78	11.45	11.98
**I1-P1**	7.91	7.78	8.54	8.48	8.33	8.92	7.25	7.12	9.41	9.87	9.26	9.92
**EW**	20.28	20.41	22.14	19.77	23.01	19.36	18.88	21.82	21.18	22.58	22.71	23.32
**PB**	6.36	6.59	7.14	6.49	7.09	6.28	5.72	6.48	6.66	7.14	7.25	7.14
**ML**	18.44	19.10	20.14	19.39	20.06	20.42	17.59	18.47	18.11	18.96	18.89	19.60
**LML**	7.01	7.27	7.43	7.54	7.61	7.35	7.74	7.47	7.32	7.07	7.38	7.78

**MR**	11.90	12.48	12.77	12.15	13.22	12.30	9.68	12.30	12.24	12.98	13.32	13.54
**RL1**	19.60	19.87	21.77	20.50	20.36	22.47	17.38	19.05	17.65	19.11	19.64	19.11
**RL2**	22.07	22.75	23.43	22.26	23.14	23.79	20.56	21.30	20.59	21.87	22.42	22.07
**I2-M4**	10.53	10.91	12.40	11.30	10.79	11.81	11.26	10.91	10.30	10.49	10.93	10.93

Table S4: PCA results for shape of cranium with variance and cumulative values to 100%.

	Eigenvalues	% Variance	Cumulative %
**1**	0.00080071	22.991	22.991
**2**	0.00054307	15.593	38.584
**3**	0.00039947	11.47	50.054
**4**	0.00029086	8.351	58.405
**5**	0.0002394	6.874	65.279
**6**	0.00017401	4.996	70.276
**7**	0.00014986	4.303	74.578
**8**	0.00012296	3.53	78.109
**9**	0.00010684	3.068	81.176
**10**	0.00008941	2.567	83.744
**11**	0.00007499	2.153	85.897
**12**	0.00006553	1.882	87.778
**13**	0.00006473	1.859	89.637
**14**	0.00005267	1.512	91.149
**15**	0.00004269	1.226	92.375
**16**	0.00003841	1.103	93.478
**17**	0.00003225	0.926	94.404
**18**	0.00002996	0.86	95.264
**19**	0.00002671	0.767	96.031
**20**	0.00002306	0.662	96.693
**21**	0.00002025	0.581	97.275
**22**	0.00001819	0.522	97.797
**23**	0.00001525	0.438	98.235
**24**	0.00001127	0.324	98.558
**25**	0.00000962	0.276	98.835
**26**	0.00000889	0.255	99.09
**27**	0.0000075	0.215	99.305
**28**	0.00000668	0.192	99.497
**29**	0.00000564	0.162	99.659
**30**	0.00000365	0.105	99.764
**31**	0.00000323	0.093	99.857
**32**	0.00000287	0.082	99.939
**33**	0.00000168	0.048	99.987
**34**	0.00000045	0.013	100
